# The Dental Student

**Published:** 1874-09

**Authors:** J. Taft


### THE DENTAL STUDENT.

BY J. TAFT.

*Read before the District Dental Society of the Seventh Judicial District of the State of New York, Rochester, N. Y., June 2d. and 3d.,* 1874.

The proper training and education of those who propose to enter the ranks of the dental profession, become its active members, sustain and develope it, are subjects of great interest and importance, not only to the student himself, but to the whole profession, and to the public as well.

The occasion for considering this subject, arises from the fact that, great imperfection exists in the education of dental students, or at least, that much more might be done than is accomplished, by the course now generally pursued.

This is true not only of private or office instruction, but of that given in the schools.

The object in discussing this subject is that we may arrive at a better apprehension of its importance, and manner of accomplishment, and be stimulated into a higher activity in this direction.

It is hardly necessary here to refer to the present status of dental education. It is quite apparent to any close observer that it is very defective; its imperfections are shown upon every hand. It is not intimated by this, that there has been any retrograde movement in this direction, however, but that much more ought to be done, in the way of elevating the standard than has yet been attempted.

*Who shall become dentists?* This question may not be easily answered in full, yet there are some thoughts bearing directly upon the subject that may be presented so as to lead somewhat in the right direction.

And first as to natural endowment, some have maintained the opinion, or professed to do so, that all are about alike endowed by nature, for the various avocations of human life. Were this true, we should expect to find more general uniformity in the execution of life's business.

Others maintain that each individual is especially fitted by nature, for some particular work or pursuit, and would prove a failure in any other direction. Now, the truth lies between these extremes. It is well to consider in what respects, and how far nature bestows special endowments.

Industry, perseverance, steadfastness of purpose and continuity of thought, though susceptible of improvement by cultivation, and may be modified by circumstances, are faculties given by nature, in very different degreesto different persons, some possessing but the faintest traces of any of these endowments, others possessing some or all of themin a marked degree.

It must also be remembered that there is a very great difference in susceptibility of cultivation, some cases yielding abundantly under the influence of well directed cultivation, while others prove almost totally barren of desirable results. A love of occupation is an important element of success, indeed, success oftentimes depends upon this. Two persons in other respects similar, but differing in their regard for a pursuit, will attain very different results.

The simple mention of this fact is sufficient to bring to mind many illustrations. Another important faculty desirable for the dental student is artistic and mechanical ability. Natural endowments in this respect are very diverse, some being highly gifted, others very poorly, indeed, with an almost infinite variety between these extremes. A facility of manipulation and execution, is of the highest importance to the dentist, a manifest deficiency in this respect, is certainly discouraging, if not a positive counter indication to entering the dental profession, though it is true that in rare instances cultivation will accomplish surprising results, in bringing out manipulative skill; but cases of this kind are so rare, that they should be relied upon with great caution.

A high degree of manipulative skill, involves a very perfect command and control by the will, and indeed by all the intellectual faculties of the various organs of the body, especially those immediately concerned in the various acts of manipulation. One speaking of the talents necessary for the physician, enumerates as the chief, "good senses and good sense." By the former he means the good and healthy condition of the physical senses, sight, hearing, touch, taste and smell. By the latter is meant ability and integrity of judgment.

Now, if these are important, necessary for the physician, they are equally so for the dentist, and if they are necessary requirements for the dentist, they can not be overlooked or disregarded in the student who proposes to become a dentist. The same writer says: "A man whom nature has made a fool, no college, no training, no discipline, can ever convert into a physician, a fool being unquestionably the worst material of which one can attempt to make a doctor of medicine, or of any other useful science."

The fitness or adaptibility of any one for a dental studentship, should always be submitted to, and decided upon by the intelligent, appreciative dentist, of integrity and of correct views of the status, and wants of the profession. Whatever other influences may lead any one to encourage or receive a dental student, the considerations already referred to, should not be disregarded.

The subject of dental studentship should be thoroughly reviewed and considered in all its aspects by the whole profession, and by concert of action attain uniformity, as nearly as possible," especially in reference to the best plan to be pursued through a course of instruction. Now and then, in the profession efforts have been made, looking in this direction.

Fifteen years ago, the Mississippi Valley Dental Society passed a resolution, by which its members bound themselves not to receive a student for a less period than two years, and they have as a rule acted up to the requirement, and many instances might be given, in illustration of the good accomplished by that resolution, in the changes that have been made in dental instruction in private offices, and by directing the attention of the profession to a very important subject, and causing the student himself, to entertain a more exalted opinion of that to which he aimed, and by eliciting the interest of the people, and increasing their knowledge and confidence.

The Michigan Dental Association, in 1870, passed the following:

"*Resolved*, that we, the members of the Michigan Dental Association, hereby pledge ourselves, each to the others, that we will not take, or allow any person in our offices as student or learner, or working under instructions, unless such person shall pledge himself to study dentistry, at least, two years under the instruction of a reputable practitioner of dentistry, and attend at least two full courses of lectures, at a dental college, and graduate before offering his services to the public as a dentist." This is perhaps the highest position that has ever been taken by any dental society in the country.

If all the reputable practitioners of the profession would adopt and live up to this resolution, such progress and improvement would be realized as have not been dreamed of, even by the most sanguine. Such resolutions indicate that there is a general conviction that natural endowments can not take the place of, nor compensate for educational attainments.

That a thorough training is necessary for the dental student, before he assumes the responsibility that attaches to the practice of his profession, will be theoretically admitted by all, but practically it is ignored by many, and by some, too, of whom better things should be expected.

A recent President of the Michigan Dental Society, in his retiring address says: "Let us endeavor, with might and main, to stimulate those who are to succeed us, to attain to far brighter and nobler heights, than has ever been our lot to reach.

No boy should ever be permitted to commence the study of dentistry, simply because he is ingenious, and has a natural taste for mechanism, this should be a consideration, but by no means the qualifications necessary to a successful student, no dentist should take into his office, or encourage a student until he has made his fitness an especial study.

The practice of taking mere boys into the office as students, before they have had any preparatory education, and expect them to make honorable, worthy and successful members of the profession is much to be deplored. No business man would take into his employ a book-keeper on the simple recommendation of his proficiency in penmanship. It will be admitted by all that the better primary education one may possess, the better he will be prepared to enter upon the study of any specialty he may desire to engage in. Do we not at all times regret that our early instruction was not more complete. I would that every student in dentistry was required to graduate from a medical college, before he should be deemed qualified to matriculate in a dental college, and I am fully convinced that the time is not very far distant, when every dentist shall be a physician, not when every physician shall be a dentist, for will it not be acknowledged by all, that dentistry is but an advanced specialty of medicine, and will be best practiced by him who has the best knowledge of medicine."

It should ever be the aim of the dental teacher to instil into the mind of his student a just apprehension and true appreciation of the objects and capabilities of the profession as well as of those things that are necessary, to enable the dentist to fulfill successfully and satisfactorily the demands that may be made upon him by suffering humanity.

No one should enter upon a course of dental study, who does not, or can not readily be made to appreciate the requirements, duties and responsibilities of the profession. Nor should any one be encouraged to enter it, whose chief aim is a mercenary one, to make money by any and every means that will be tolerated. Money is a good thing, desirable and necessary, but there are other things better, more sacred, that should not be prostituted to the morbid desire for gain. No one in whom this spirit predominates should be encouraged to enter the dental profession.

In conclusion, let the dental student be a man of a good degree of intellectual ability, faithfulness and integrity of purpose, for the accomplishment of the highest good, industry, perseverance. A good preliminary education, and possess a sound mind, in a sound and well attuned body.

